# Sars-CoV-2 Envelope and Membrane Proteins: Structural Differences Linked to Virus Characteristics?

**DOI:** 10.1155/2020/4389089

**Published:** 2020-05-30

**Authors:** Martina Bianchi, Domenico Benvenuto, Marta Giovanetti, Silvia Angeletti, Massimo Ciccozzi, Stefano Pascarella

**Affiliations:** ^1^Department of Biochemical sciences “A Rossi Fanelli”, Sapienza University of Rome, 00185 Rome, Italy; ^2^Unit of Medical Statistics and Molecular Epidemiology, University Campus Bio-Medico of Rome, Rome, Italy; ^3^Flavivirus Laboratory, Oswaldo Cruz Institute, Oswaldo Cruz Foundation, Rio de Janeiro, Brazil; ^4^Unit of Clinical Laboratory Science, University Campus Bio-Medico of Rome, Rome, Italy

## Abstract

The Coronavirus Disease 2019 (COVID-19) is a new viral infection caused by the severe acute respiratory coronavirus 2 (SARS-CoV-2). Genomic analyses have revealed that SARS-CoV-2 is related to Pangolin and Bat coronaviruses. In this report, a structural comparison between the Sars-CoV-2 Envelope and Membrane proteins from different human isolates with homologous proteins from closely related viruses is described. The analyses here reported show the high structural similarity of Envelope and Membrane proteins to the counterparts from Pangolin and Bat coronavirus isolates. However, the comparisons have also highlighted structural differences specific of Sars-CoV-2 proteins which may be correlated to the cross-species transmission and/or to the properties of the virus. Structural modelling has been applied to map the variant sites onto the predicted three-dimensional structure of the Envelope and Membrane proteins.

## 1. Introduction

COVID-19 has become a planetary emergency which is seriously threatening human health [1, 2]. Development of effective therapeutic and prevention strategies is significantly hampered also by the lack of detailed structural information on virus proteins, although several crystallographic structures of Sars-CoV-2 proteins are now available [3–5]. In this report, a structural comparison between the Sars-CoV-2 surface proteins from different isolates with homologous proteins from closely related viruses such as those from Bat and Pangolin is described. This work has been focussed onto the Envelope (E) and Membrane (M) proteins that, along with the Spike, form the virus protein interface to the external environment. The Spike glycoprotein has been already extensively studied, and a few crystallographic structures are available in the Protein Data Bank [3–6]; consequently, this protein has not been specifically addressed within this note. Identification of local structural differences, even minimal, to the closest virus proteins may indicate the mutations that enabled Sars-CoV-2 to cross species and/or to acquire its peculiar pathogenic properties [7, 8]. Indeed, a number of examples have been reported in the scientific literature suggesting how even single point mutations in virus proteins can significantly alter their biology and pathogenesis [9, 10]. Therefore, comparative studies may shed light on the molecular mechanisms through which an epidemic of epizootic origin can emerge and may also suggest molecular targets for therapeutics or reverse vaccinology experiments.

## 2. Material and Methods

Nucleotide and protein sequences have been taken from GenBank [11] data repository. Blast suite [12] has been used for databank searches; Jalview [13] and MAFFT [14] have been used for multiple sequence display and alignment, respectively. Transmembrane helix prediction has been obtained by TMHMM [15], MEMSAT [16], and Protter [17]. Cd-hit program [18] has been used for sequence clustering. Homology modelling relied on Swiss-Model [19], Modeller [20], or HHpred [21] and structure display and analysis on Open-Source PyMOL [22]. When necessary, I-Tasser [23] has been used as an alternative source of ab initio homology models.

## 3. Results

### 3.1. Databank Searches and Structure Modelling

From the GenBank repository, 797 complete genomes of Sars-CoV-2 have been collected (the full list is reported in Supplementary Data). The TblastN program has been used to extract the sequences of E and M proteins from each genome. To remove redundancy within each E and M protein set, cd-hit clustering has been applied at 100% sequence identity level: identical sequences have been assigned to the same group for which only one representative has been considered for further analysis. The Sars-CoV-2 E and M protein sets have been grouped into three and seven clusters, respectively. This finding suggests that within the 797 genomes three and seven variants of the E and M proteins can be observed, respectively. E and M homologous proteins from closely related virus have been retrieved from the GenBank using the TblastN tool.

### 3.2. Envelope Protein

**Figure 1:**
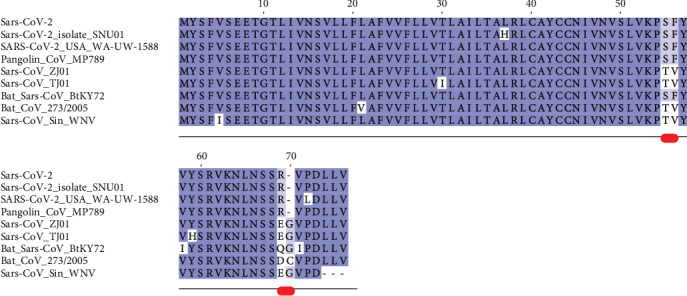
Multiple sequence alignment among Sars-CoV-2 Envelope protein variants and a set of the most similar homologous proteins. The sequence labelled Sars-CoV-2 corresponds to the reference sequence identified by the RefSeq code YP_009724392. Red lines below the alignment indicate the changed sites discussed in the text. Blu background denotes conserved alignment positions.

**Figure 2:**
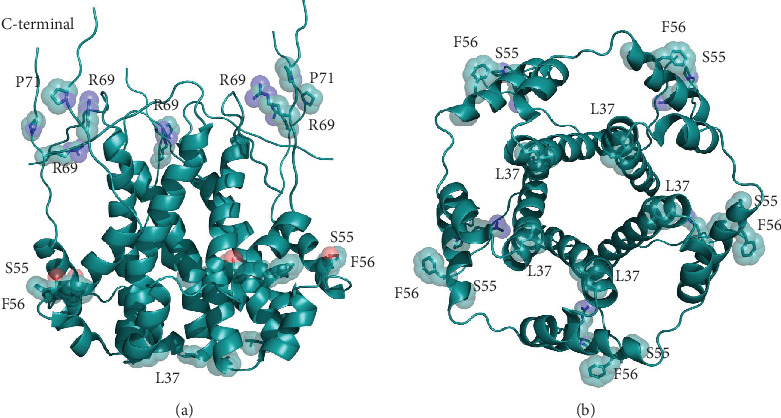
Three-dimensional model of the viroporin-like tetrameric assembly of the E protein from Sars-CoV-2 represented as a cartoon model. Residues corresponding to the mutated sites indicated in Figure 1 are displayed as transparent space-filling spheres and labelled with the amino acid one-letter code. The C-terminal segments of the model are reported for completeness. However, they convey no structural information due to lack of a corresponding segment in the structural template used in homology modelling. Structure in panel (b) is rotated by approximately 180° along the *x* axis with respect to the orientation shown in panel (a).

The E protein is conserved across *β*-coronaviruses. Only three variants have been found in the Sars-CoV-2 E set collected. Sequence comparisons show that the Sars-CoV-2 E protein from the reference genome (RefSeq code YP_009724392) is identical to the sequences from Pangolin CoV MP798 and Bat CoV CoVZXC21, CoVZC45, and RaTG13 isolates. The multiple sequence alignment reported in Figure 1 demonstrates that a distinguishing feature of Sars-2-CoV E variants is the presence of Arg at position 69 that substitutes Glu, Gln, Asp in other homologous Sars-CoV E proteins. This site is followed by a deletion in position 70 corresponding to Gly or Cys in the other proteins. Sars-CoV-2 E sequences differ from the homologous proteins also at positions 55-56, where the dyad Ser-Phe replaces Thr-Val (except in Bat coronavirus isolate BtKY72, accession code KY352407). Variants of the Sars-CoV-2 E protein differ at positions 37 and 72 where His substitutes a Leu and Leu replaces a conserved Pro, respectively. The size of each Envelope variant cluster is reported in Table 1 along with accession codes and definitions of the isolates. A homology model of the E protein has been built with Modeller using as a template the pentameric ion channel structure of Sars-CoV protein identified by the PDB code 5X29. This sequence shares 91% identity to Sars-CoV-2 E protein and covers the segment encompassed by positions 8-65.  Figure 2 displays the structure of the homology model of the Sars-CoV-2 E protein assembled as a pentameric viroporin-like protein.  Figure 2 displays also the position of the variant sites onto the three-dimensional model. Prediction of the transmembrane helices is difficult in a short protein. Therefore, transmembrane topology cannot be assigned reliably. Likewise, experiments have not clarified definitively which portions of the E protein are exposed to the external or internal side of the virus membrane [24].

### 3.3. Membrane Glycoprotein

**Figure 3:**
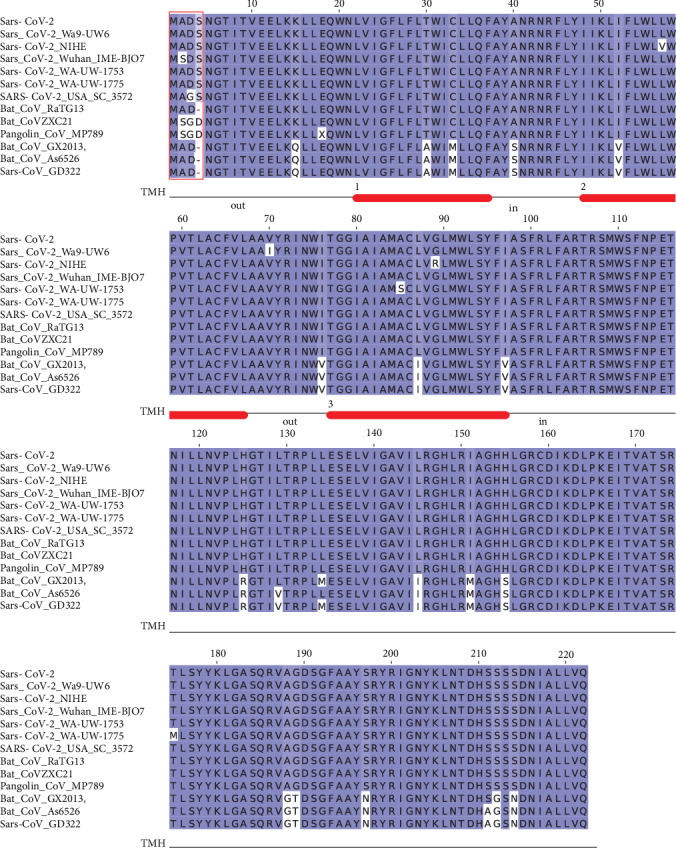
Multiple sequence alignment among Sars-CoV-2 M protein variants and a set of most similar homologous proteins. The sequence label Sars-CoV-2 indicates the reference sequence identified by the RefSeq code YP_009724393. Red box indicates the variant sites at the N-terminal discussed in the text. Numbered red bars under the multiple alignment mark the prediction of transmembrane helices. The location of the connect loop with respect to the virion surface is indicated as “in” or “out”. Blu background denotes conserved alignment positions.

The M glycoprotein is conserved across the *β*-coronaviruses. However, seven variants of Sars-CoV-2 M protein were identified in the collected set, while only three variants were observed for the E protein (Figure 3). The multiple sequence alignment shows a remarkable similarity (98% identity) among the Sars-CoV-2 M variants and the sequences from Bat and Pangolin isolates. However, a difference at the N-terminal position (Figure 3) can be observed: the insertion of a Ser residue at position 4 of human Sars-CoV-2 seems to be a unique feature of this protein. In the corresponding position, the RaTG13 Bat M protein displays a deletion, while Bat CoVZXC21, CoVZC45, and Pangolin MP789 proteins have an Asp residue. The seven M protein variants differ at positions 2, 3, 57, 70, 85, 89, and 175. The size of each Membrane variant cluster is reported in Table 1 along with accession codes and definitions of the isolates. Noteworthy, the protein from the Sars-CoV-2 NIHE isolate (accession code MT127115) possesses an Arg instead of a conserved Gly at position 89 (Figure 3). The mutation occurs within a predicted transmembrane helix and, if confirmed, may have a significant impact on the protein properties (Figure 3).

**Figure 4:**
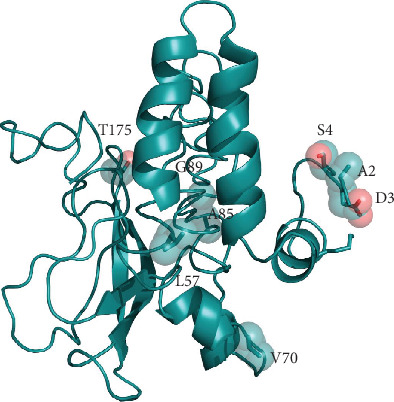
I-Tasser model of the Membrane protein represented as cartoon model. Variant positions are displayed as transparent space-filling spheres and labelled with the amino acid one-letter code.

The three-dimensional model of the M protein has been taken from the I-Tasser server (code QHD43419) as other methods failed to find any suitable template. However, it should be mentioned that HHpred found a weak local affinity, albeit below the statistical significance level, to 4N31, a peptidase-like protein from *Streptococcus pyogenes* essential for pilus polymerisation.  Figure 4 displays the positions of the variant sites onto the model structure. This model has been predicted by ab initio techniques. Therefore, it should be considered with great caution and should be treated as a low-resolution approximation of the real structure. According to the prediction of the transmembrane helix topology, the N- and C-terminal portions of the M protein are exposed outside and inside the virus particle, respectively (Figure 4).

## 4. Discussion

Previous studies pointed out that E and M proteins could be important for viral entry, replication, and particle assembly within the human cells [24, 25]. According to the accepted theories, the current COVID-19 pandemic has been caused by the cross-species transmission of a *β*-coronavirus normally hosted by Bats and, perhaps, Pangolin to humans [3, 26]. In this paper, E and M proteins from 797 Sars-CoV-2 genomes have been compared to the counterparts taken from the most closely related virus also to evaluate the potential role of amino acid mutations in the epizootic origin of COVID-19. E protein is a minor component of the virus membrane though it is deemed to be important for many stages of virus infection and replication [24, 25]. Sequence comparison has shown that this protein is identical to the counterparts of specific Bat and Pangolin coronavirus isolates, even though the Sars-CoV-2 sequence seems to possess specific modifications and characteristics with respect to other Sars CoVs. In particular, Arg69, a positively charged amino acid, replaces Glu or Gln residues, negatively charged and neutral, respectively, in the homologous CoV proteins. Moreover, a deletion specific to Sars-CoV-2 proteins flanks this position. Unfortunately, it is not possible to predict reliably whether the sites of these modifications are exposed to the internal or external side of the membrane. In any case, the substitution and the deletion appear a rather drastic change and may have a significant impact on conformational properties and possibly on protein-protein interactions. Further structural studies are needed. However, it may be hypothesized that these changes can also affect the oligomerization process necessary to form a transmembrane ion channel.

It has been demonstrated that M protein is more prevalent within the virus membrane, and it is deemed to be important for the budding process of coronaviruses. Indeed, during the process of virus particle assembly, this protein interacts with the Nucleocapsid, Envelope, Spike, and Membrane glycoprotein itself [25]. Moreover, in Alphacoronaviruses, it has been demonstrated that this protein cooperates with the Spike during the cell attachment and entry [27]. Therefore, mutations occurring at the N-terminus region, which is exposed to the virus surface, could play a key role in the host cell interaction.

In conclusion, the analyses here reported show the structural similarity of E and M proteins to the counterparts from Pangolin and Bat coronavirus isolates. At the same time, comparisons have highlighted structural differences specific of Sars-CoV-2 proteins which may be correlated to the cross-species transmission and/or to the properties of the virus. Although further studies are needed, it is clear that these amino acid variations have been important for the virus evolutionary history, and the results may hint at how similar mutations within the coronavirus family can lead in the next years to other epizootic epidemic events similar to the one that we are experiencing these days.

## Figures and Tables

**Table 1:** Size of the variant clusters of the Sars-CoV-2 Envelope and Membrane proteins.

Variant	Cluster size (no. of sequences)	Accession code	Definition
**Envelope**			
YP_009724392 (reference)	795		

His37	1	MT03980	Korea/SNU01/2020

Leu72	1	MT293206	USA/WA-UW-1588/2020

**Membrane**			
YP_009724393 (reference)	773		

Ser2	1	MT291836	CHN/Wuhan_IME-BJ07/2020

Gly3	1	MT325626	USA/SC_3572/2020

Val57, Arg89	1	MT127115	VIE/NIHE/2020

		MT293184	USA/WA-UW-1297/2020
lle70	3	MT326166	USA/WA-UW-1735/2020
		MT293211	USA/WA-UW-1591/2020

Ser85	1	MT326167	USA/WA-UW-1753/2020

		MT326093	USA/WA-UW-1775/2020
Met175	2	MT246451	USA/WA-UW-194/2020

## Data Availability

All sequence data are available in the GenBank repository. The complete list is available in the Supplementary Materials.

## Supplementary Materials

Supplementary Materials The supplementary data consist of an Excel file containing the list of the 797 Sars-CoV-2 genomes retrieved from GenBank and analysed in the article. For each genome, the sequence header is displayed as found in the GenBank. The header reports the accession code in the first field of each line after the “>” character.
