# Supplementary material for: Sars-CoV-2 Envelope and Membrane Proteins: Structural Differences Linked to Virus Characteristics?
Source: Biomed Res Int. 2020 May 30;2020:4389089. doi: 10.1155/2020/4389089 (PMC7261327; doi:10.1155/2020/4389089)
Supplement: Supplementary Materials — The supplementary data consist of an Excel file containing the list of the 797 Sars-CoV-2 genomes retrieved from GenBank and analysed in the article. For each genome, the sequence header is displayed as found in the GenBank. The header reports the accession code in the first field of each line after the “>” character. [file 4389089.f1.zip › Supplementary_description.pdf]

Sars-CoV-2 Envelope and Membrane proteins: structural differences linked to virus characteristics?

Martina Bianchi[1], Domenico Benvenuto[2], Marta Giovanetti[3], Silvia Angeletti[4], Massimo Ciccozzi[2] and Stefano Pascarella[1*]

[1]Department of Biochemical sciences "A Rossi Fanelli", Sapienza University of Rome, 00185 Rome, Italy
[2]Unit of Medical Statistics and Molecular Epidemiology, University Campus Bio-Medico of Rome, Rome, Italy.
[3]Flavivirus Laboratory, Oswaldo Cruz Institute, Oswaldo Cruz Foundation, Rio de Janeiro, Brazil.
[4]Unit of Clinical Laboratory Science, University Campus Bio-Medico of Rome, Rome, Italy

Supplementary Data Description

Table 1 displays the number of sequences contained in each cluster of Envelope and Membrane protein variants as determined by the application of cd-hit at 100% sequence identity threshold. The accession codes and definitions of isolates of the smaller clusters have been reported. The sum of the numbers of all isolates of Sars-CoV-2 Membrane proteins does not equal 797, the number of initial genomes considered, because sequences containing ambiguous codons translated as "X" have been removed by cd-hit.

The **Excel file** contains the list of the 797 Sars-CoV-2 genomes retrieved from GenBank and analysed in the article. For each genome, the sequence header is displayed as found in the GenBank. The header reports the accession code in the first field of each line after the ">" character.
